# Embryonic Hematopoietic Progenitor Cells Reside in Muscle before Bone Marrow Hematopoiesis

**DOI:** 10.1371/journal.pone.0138621

**Published:** 2015-09-21

**Authors:** Yuka Tanaka, Tomoko Inoue-Yokoo, Kasem Kulkeaw, Chiyo Yanagi-Mizuochi, Senji Shirasawa, Yoichi Nakanishi, Daisuke Sugiyama

**Affiliations:** 1 Center for Advanced Medical Innovation, Kyushu University, Fukuoka, Japan; 2 Department of Cell Biology, Faculty of Medicine, Fukuoka University, Fukuoka Japan; 3 Department of Research and Development of Next Generation Medicine, Kyushu University Faculty of Medical Sciences, Fukuoka, Japan; 4 Center for Clinical and Translational Research, Kyushu University Hospital, Fukuoka, Japan; National Institutes of Health, UNITED STATES

## Abstract

In mice, hematopoietic cells home to bone marrow from fetal liver prenatally. To elucidate mechanisms underlying homing, we performed immunohistochemistry with the hematopoietic cell marker c-Kit, and observed c-Kit(+) cells localized inside muscle surrounding bone after 14.5 days post coitum. Flow cytometric analysis showed that CD45(+) c-Kit(+) hematopoietic cells were more abundant in muscle than in bone marrow between 14.5 and 17.5 days post coitum, peaking at 16.5 days post coitum. CD45(+) c-Kit(+) cells in muscle at 16.5 days post coitum exhibited higher expression of *Gata2*, among several hematopoietic genes, than did fetal liver or bone marrow cells. Colony formation assays revealed that muscle hematopoietic cells possess hematopoietic progenitor activity. Furthermore, *exo utero* transplantation revealed that fetal liver hematopoietic progenitor cells home to muscle and then to BM. Our findings demonstrate that hematopoietic progenitor cell homing occurs earlier than previously reported and that hematopoietic progenitor cells reside in muscle tissue before bone marrow hematopoiesis occurs during mouse embryogenesis.

## Introduction

In mice, the site of embryonic hematopoiesis changes over an approximately 20-day gestation period. Primitive hematopoiesis begins in the yolk sac (YS), producing mainly primitive erythroid cells at 7.5 days post coitum (dpc). This process is transient and decreases by 12.5 dpc [1]. Adult-type hematopoiesis, termed definitive hematopoiesis, is characterized by the appearance of cells with definitive erythroid, lymphoid and hematopoietic stem cell (HSC) potentials. Definitive myelo-erythroid progenitor cells appear in the YS around 8.25 dpc and are then seeded to fetal liver (FL) [2]. HSCs are likely generated in the YS, intra-embryonic para-aortic-splanchnopleural mesoderm/Aorta-Gonad-Mesonephros (AGM) region, and placenta [3–7]. Previously, we reported that circulating c-Kit-positive hematopoietic cells (HCs) home to FL [8]. Both morphological observation and *in vitro* experiments indicated that FL itself does not produce hematopoietic stem/progenitor cells (HSPCs) but is rather colonized by HCs originating elsewhere after 9.5 dpc [9–12]. Taken together, HSPCs likely circulate and home to FL, where their number dramatically increases as definitive erythropoiesis occurs extensively at mid-gestation [11–13]. After HSC expansion in FL, HSCs home to the fetal spleen, where they differentiate from 13.5 to 14.5 dpc [14]. As HSCs with reconstitution ability are first detected in bone marrow (BM) at 17.5 dpc, they likely home to this site to start life-long hematopoiesis [15].

It remains unclear why hematopoietic sites dramatically shift during embryogenesis. Previously, we demonstrated that Dlk-1-positive hepatoblasts function as niche cells to regulate HSC homing and differentiation by secretion of extra-cellular matrix (ECM) proteins and cytokines, such as erythropoietin (Epo) and stem cell factor (SCF) [16, 17]. ECMs, which typically function in cell adhesion, cell-to-cell communication and differentiation, often partner with integrins in these processes [18–20]. In FL of beta-1 integrin (fibronectin receptor beta, CD29) knockout chimeric embryos, beta-1 integrin-positive HCs homed to the FL, while those lacking beta-1 integrin did not [19, 21]. We also demonstrated that HSPCs and erythroid cells in FL express beta-1 integrin, while circulating erythroid cells do not, suggesting that beta-1 integrin regulates FL homing [21, 22]. The ECM protein fibronectin is a beta-1 integrin ligand and reportedly promotes homing ability of HCs *in vitro* [22]. Given that fibronectin is highly expressed in FL, it likely regulates homing of HCs expressing beta-1 integrin.

Although mechanisms underlying HC homing to FL from the circulation have been investigated, how cells home from the FL to embryonic BM is not fully understood. Fetal BM forms by 15.5 dpc [15], but HSC activity is not detected there until 17.5 dpc, suggesting that HSCs remain in the FL or other tissues. Here, to investigate mechanisms underlying fetal BM homing, we performed immunohistochemistry of embryonic bones and surrounding tissues. We observed c-Kit-positive HCs residing in muscle tissue surrounding bones late in gestation. In addition, muscle HCs showed HPC ability, as determined by colony formation assays. These findings suggest that HPCs reside in muscle tissue before homing to the fetal BM.

## Materials and Methods

### Mice

C57BL/6 mice (Nihon SLC, Hamamatsu, Japan and Kyudo, Tosu, Japan) and enhanced green fluorescence (EGFP) Tg mice (Research Institute for Microbial Diseases, Osaka University, Osaka, Japan) were used in this study. Animals were handled according to Guidelines for the Care and Use of Laboratory Animals of Kyushu University. This study was approved by the Animal Care and Use Committee, Kyushu University (Approval ID: A25-119-1).

### Cell preparation

Left and right femurs and muscle tissues surrounding those structures of 14.5 to 19.5 dpc C57BL/6 mouse embryos were used to obtain single cell suspensions. Tissues were trimmed from femurs and incubated with 3 mg/ml collagenase in medium containing 10% fetal bovine serum (FBS) for 20 minutes at 37°C. Cells were then filtered through 70 μm nylon cell strainers (BD Biosciences, San Jose, CA). BM cells were flushed out with PBS containing 2% FBS using 29 to 32G needles with syringes (TERUMO, Tokyo, Japan) and filtered through 40 μm nylon cell strainers (BD Biosciences). FLs from 14.5 dpc and 16.5 dpc embryos were dissected out, and single cell suspensions were prepared by digesting tissues with 3 mg/ml collagenase in medium containing 10% FBS for 20 minutes at 37°C. Cells were then filtered through 40 μm nylon cell strainers (BD Biosciences). BM cells from femurs and tibias of 3-month-old adult C57BL/6 mice were dissected out and then flushed out with PBS containing 2% FBS from 27G needles and syringes (TERUMO). Cells were then filtered through 40 μm nylon cell strainers (BD Biosciences). Fetal blood was obtained from 16.5 dpc embryos. Blood cells were then washed 3 times with PBS containing 2% FBS.

### Flow cytometric analysis and cell sorting

To avoid non-specific antibody binding, cells were treated with anti-mouse CD16/32 Fc binding blocker (eBioscience, San Diego, CA) for 10 minutes at 4°C. After one PBS wash, cells were stained with PE-conjugated anti-mouse CD45 antibody, PE-Cy7-conjugated anti-mouse c-Kit antibody, FITC-conjugated anti-mouse Sca-1 antibody and APC-Cy7-conjugated anti-mouse Mac-1 (CD11b) antibody (all from Biolegend, San Diego, CA). Pacific Blue-conjugated anti-mouse F4/80 antibody (Biolegend) was used to remove macrophages from CD45(+) c-Kit(+) cells in muscle tissue surrounding femurs. To further characterize CD45(+) c-Kit(+) Sca-1(+) cells, biotin-conjugated anti-mouse CD150 antibody (Biolegend), anti-mouse CD34 antibody (Biolegend) and anti-mouse EPCR antibody (Biolegend) were used for staining and were then detected using V500-conjugated streptavidin (BD Bioscience). To analyze lineage marker expression, cells were stained with biotin-conjugated anti-mouse Ter119, Gr-1, CD4, CD8, B220 and Mac-1 or F4/80 antibodies, followed by detection using APC-Cy7-conjugated streptavidin (all from Biolegend). Cells sorted with BD FACS Aria (BD Bioscience) were collected into RNAlater^®^ (Life Technologies, Carlsbad, CA) for gene expression analysis. Cells used for morphological analysis were sorted into medium containing 10% FBS and stained using May-Grünwald Giemsa staining.

### Immunohistochemistry

Femurs and surrounding muscle tissues of 14.5 to 19.5 dpc embryos were dissected and fixed in 2% paraformaldehyde (PFA) in PBS at 4°C for overnight. Fixed tissues were then washed 3 times using PBS, equilibrated with 30% sucrose in PBS, embedded in OCT compound (SAKURA, Tokyo, Japan) and frozen in liquid nitrogen. Tissues were cut into 20 μm slices using a Leica CM1900 UV cryostat (Leica Microsystems, Tokyo, Japan), transferred to glass slides (Matsunami glass, Osaka, Japan) and dried thoroughly. Sections were blocked with PBS containing 1% BSA for 30 minutes and incubated at 4°C overnight with appropriate dilutions of primary antibodies: goat anti-mSCF R/c-Kit (1:400, R&D Systems, Minneapolis, MN) and rat anti-mouse CD31 (1:300, BD Bioscience). Serial sections were incubated at 4°C overnight with appropriate dilutions of the following primary antibodies: goat anti-mSCF R/c-Kit (1:400, R&D systems), hamster anti-mouse CD31 (1:400, Chemicon international, Temecula, USA) and rat anti-mouse CD45 (1:300, Biolegend). Sections were washed 3 times in PBS and incubated at room temperature for 30 minutes with appropriate dilutions of secondary antibodies: donkey anti-goat IgG AlexaFluor488 (1:400, Invitrogen, San Diego, CA), donkey anti-rat IgG Dylight549 (1:400, Jackson ImmunoResearch Laboratories, West Grove, PA) and TOTO-3 iodide (1:1500, Invitrogen), or with donkey anti-goat IgG AlexaFluor 633 (1:400, Invitrogen), rabbit anti-hamster IgG Dylight 549 (1:400, Jackson Immuno Research Laboratories), donkey anti-rat AlexaFluor 488 and/or DAPI (300 nM, Invitrogen). Sections were then washed 3 times with PBS, mounted on coverslips with fluorescent mounting medium (Dako Corporation, Carpinteria, CA) and assessed using a Fluo View 1000 confocal microscope (Olympus, Tokyo, Japan).

### Immunocytochemistry

Sorted cells were attached to glass slides (Matsunami glass) by CytoSpin4 (Thermo Fisher scientific, Waltham, MA) at 450 rpm for 7 minutes and dried thoroughly. Cells were fixed in 1% PFA at room temperature for 30 minutes. After washing 3 times with PBS, cells were blocked with PBS containing 1% BSA and 0.05% Triton X-100 at room temperature for 1 hour and then incubated overnight at 4°C with rat anti-mouse Ki-67 (1:500, Dako Corporation) primary antibody. After 3 PBS washes, cells were incubated with donkey anti-rat IgG AlexaFluor488 (1:400, Invitrogen) and TOTO-3 iodide (1:1500, Invitrogen) at room temperature for 30 minutes. After 3 PBS washes, cells were mounted on coverslips with fluorescent mounting medium (Dako Corporation) and assessed using a Fluo View 1000 confocal microscope (Olympus).

### RNA extraction and real-time polymerase chain reaction (PCR)

Total RNA was extracted from sorted cells using an RNAqueous^®^4PCR Kit (Ambion, Austin, TX) or RNAqueous^®^Micro Kit (Ambion), and mRNA was reverse-transcribed into cDNA using a High-Capacity RNA-to-cDNA^TM^ Kit (Life Technologies). Expression of *Gata2*, *Tal1*, *Mecom*, *Myb*, *Runx1*, *Myc* and *Ccnd1* was assessed by StepOnePlus^TM^ real-time PCR (Life Technologies) with TaqMan^®^Gene Expression Assays (Life Technologies). mRNA levels were normalized to *β-actin* mRNA, and relative amounts of each gene were calculated using a relative standard curve method.

### Colony formation and high proliferative potential colony forming cell (HPP-CFC) assays

Sorted cells were suspended in 4 ml of MethoCult^®^GF M3434 (STEMCELL Technologies, Vancouver, Canada), distributed into three 35-mm dishes and incubated in 5% CO_2_ at 37°C. Colonies were counted on day 14 and categorized as CFU-G (CFUs of granulocytes), CFU-M (CFUs of macrophages), CFU-GM (CFUs of granulocytes and macrophages), CFU-Mk (CFUs of megakaryocytes), and CFU-GEMM (CFUs of granulocytes, erythrocytes, monocytes and macrophages). The number of HPP-CFCs, based on the number of colonies of diameter >2 mm, was determined on day 21 [23].

### OP9/OP9 Delta1 co-culture

OP9/OP9 Delta1 cell lines were maintained in α-MEM medium (Wako Pure Chemical Industries, Osaka, Japan) supplemented with 20% FBS and 0.1% penicillin/streptomycin at 37°C in 5% CO_2_. Cells were passaged every 3–4 days. For co-culture experiments, muscle tissue CD45(+) c-Kit(+) cells obtained from 16.5 dpc C57BL/6 mouse embryos were suspended in α-MEM medium supplemented with 20% FBS, 5 ng/ml Flt3-L (PEPROTECH, NJ, USA), 1 ng/ml IL-7 (PEPROTECH) and 0.1% penicillin/streptomycin and cultured with either OP9 or OP9 Delta1 cell lines. Cells were passaged every 3–4 days. On day 16, surface expression of CD19 and B220 (B lymphoid markers) was analyzed in the case of cells cultured with the OP9 line, while surface expression of the T lymphoid markers CD4 and CD8 was analyzed for cells cultured with OP9 Delta1 line by flow cytometry.

### Organ culture

Organ culture of fetal muscle tissue was performed as reported previously with minor modifications [5]. Muscle tissues surrounding the femur at 16.5 dpc were placed onto sterilized 0.65 μm filter membranes (Millipore, Bedford, UK) and cultured in StemSpan (STEMCELL Technologies) supplemented with 0.1% penicillin/streptomycin at 37°C in 5% CO_2_. After 72 hours, tissues were collected and single cell suspensions were prepared by incubation in 3 mg/ml collagenase in medium containing 10% FBS for 15 minutes at 37°C. Colony formation assays were then performed as described above.

### *Exo utero* surgical transplantation

C57BL/6 pregnant mice at 14.5 dpc and 16.5 dpc were anaesthetized using 1.0% isoflurane and an animal anesthetizer device (MK-AT210D, Muromachi Kikai Co., LTD, Tokyo, Japan). Mice were then placed on the plate warmed to 37°C, their abdominal area was shaved, and the skin and abdominal wall were incised. The uterine wall was then cut on the side opposite the placenta. Glass needles were prepared from glass capillary tubes (Narishige, Tokyo, Japan) using a micropipette puller (PN-30, Narishige). Under a microscope, 3 μl of a cell suspension containing 1.4×10^5^ FL EGFP(+) CD45(+) c-Kit(+) cells obtained at 14.5 dpc or 5×10^3^ muscle tissue EGFP(+) CD45(+) c-Kit(+) cells obtained at 16.5 dpc (both from EGFP Tg mouse embryos) were transplanted into corresponding tissues of C57BL/6 recipient embryos. In some experiments, FL CD45(+) c-Kit(+) cells obtained from C57BL/6 mouse embryos at 14.5 dpc were labeled using a Qtracker^®^ 585 Cell Labeling Kit (Ambion), according to the manufacturer’s protocol, and 1.4×10^5^ cells were transplanted into corresponding tissues of C57BL/6 recipient embryos. The abdominal wall and skin were then sutured using 6/0 silk (Teleflex, Philadelphia, PA). After 24 or 72 hours, embryos were collected and analyzed for EGFP expression or Qdot585 fluorescence in muscle tissue and BM by flow cytometry and/or immunohistochemistry.

### Statistical analysis

Results are expressed as the mean ± standard deviation (SD) in all analyses. Paired samples were compared using Student’s *t*-test.

## Results

### Characterization of hematopoietic progenitor cells in muscle tissue surrounding embryonic femur

**Fig 1:**
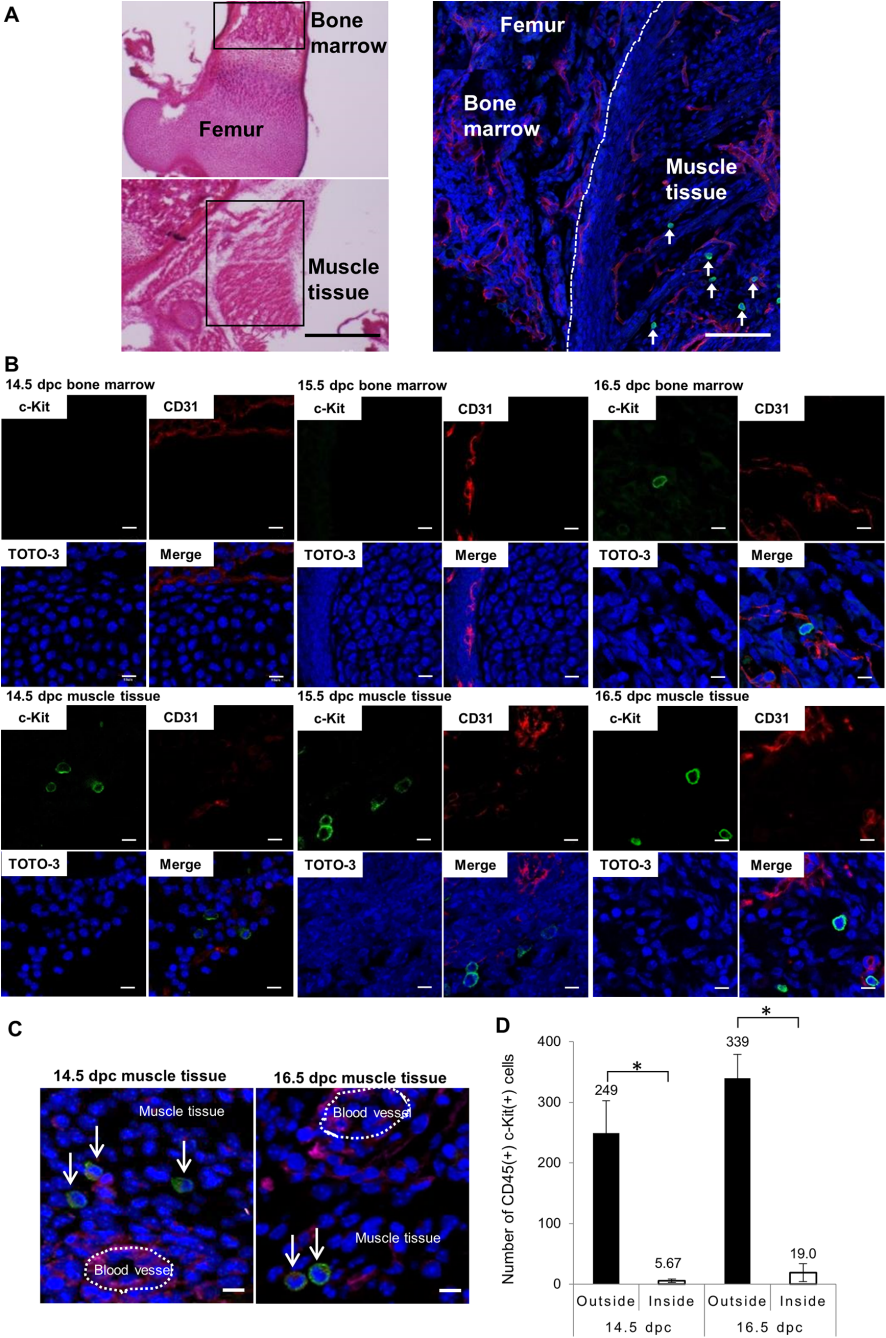
Immunohistochemistry analysis of c-Kit(+) cells in bone marrow and muscle tissue surrounding femurs. Cryosections of femurs and surrounding muscle tissues from C57BL/6 mouse embryos at 14.5 to 19.5 dpc co-stained with c-Kit as an HPC marker and CD31 as an endothelial cell marker and observed by confocal microscopy. (A) Representative images showing hematoxylin-eosin (left) and immunohistochemical (right) staining of femur and surrounding muscle tissue at 16.5 dpc. Shown is c-Kit (green), CD31 (red) and TOTO-3 iodide (blue) staining. Scale bar represents 500 μm (left) and 50 μm (right). Green c-Kit(+) cells are observed in muscle tissue and indicated by arrows in right panel. (B) Confocal images of femur and surrounding muscle tissues at 14.5 dpc, 15.5 dpc and 16.5 dpc. Shown is c-Kit (green), CD31 (red) and TOTO-3 iodide (blue) staining. Scale bar represents 10 μm for all panels. (C) Localization of CD45(+) c-Kit(+) cells in serial sections (10 μm) of muscle tissue co-stained with antibodies against CD45 (red), c-Kit (green), CD31 (magenta) and DAPI (blue) at 14.5 dpc (left) and 16.5 dpc (right). Scale bar represents 10 μm for all panels. Arrows indicate CD45(+) c-Kit(+) cells, most located in muscle tissue rather than inside blood vessels. (D) The number of CD45(+) c-Kit(+) cells inside or outside of blood vessels in two sets of femur-surrounding muscle tissues was determined by microscopic observation of serial sections. Bars represent mean values and SD of cell numbers from three individual embryos. *p<0.01.

To evaluate migration properties of HPCs, we investigated their localization in late-gestation mice. We carried out immunohistochemistry using cryosections of fetal femur and surrounding tissues at 14.5 to 19.5 dpc. Hematoxylin-eosin staining indicated normal structure of the femur BM cavity (Fig 1A, upper left) and surrounding fibriform muscle tissues at 16.5 dpc (Fig 1A, lower left). Cryosections were co-stained with c-Kit as an HPC marker and CD31 as an endothelial cell marker (Fig 1A, right and 1B). As shown in Fig 1B, round c-Kit(+) cells were seen in muscle tissue surrounding femurs, whereas c-Kit(+) cells were rarely observed in BM from 14.5 to 16.5 dpc. c-Kit(+) cells were also observed in other areas of muscle tissue (S1 Fig). Analysis of adult mice using immunohistochemistry and flow cytometry showed no evidence of c-Kit(+) cells in adult muscle tissue (S2A and S2B Fig). Examination of serial sections of muscle tissue at 14.5 dpc and 16.5 dpc revealed that most CD45(+) c-Kit(+) cells resided in muscle tissue (Fig 1C). As shown in Fig 1D, the number of CD45(+) c-Kit(+) cells was greater outside of blood vessels in muscle tissue than inside (43.9-fold greater at 14.5 dpc and 17.8-fold greater at 16.5 dpc).

**Fig 2:**
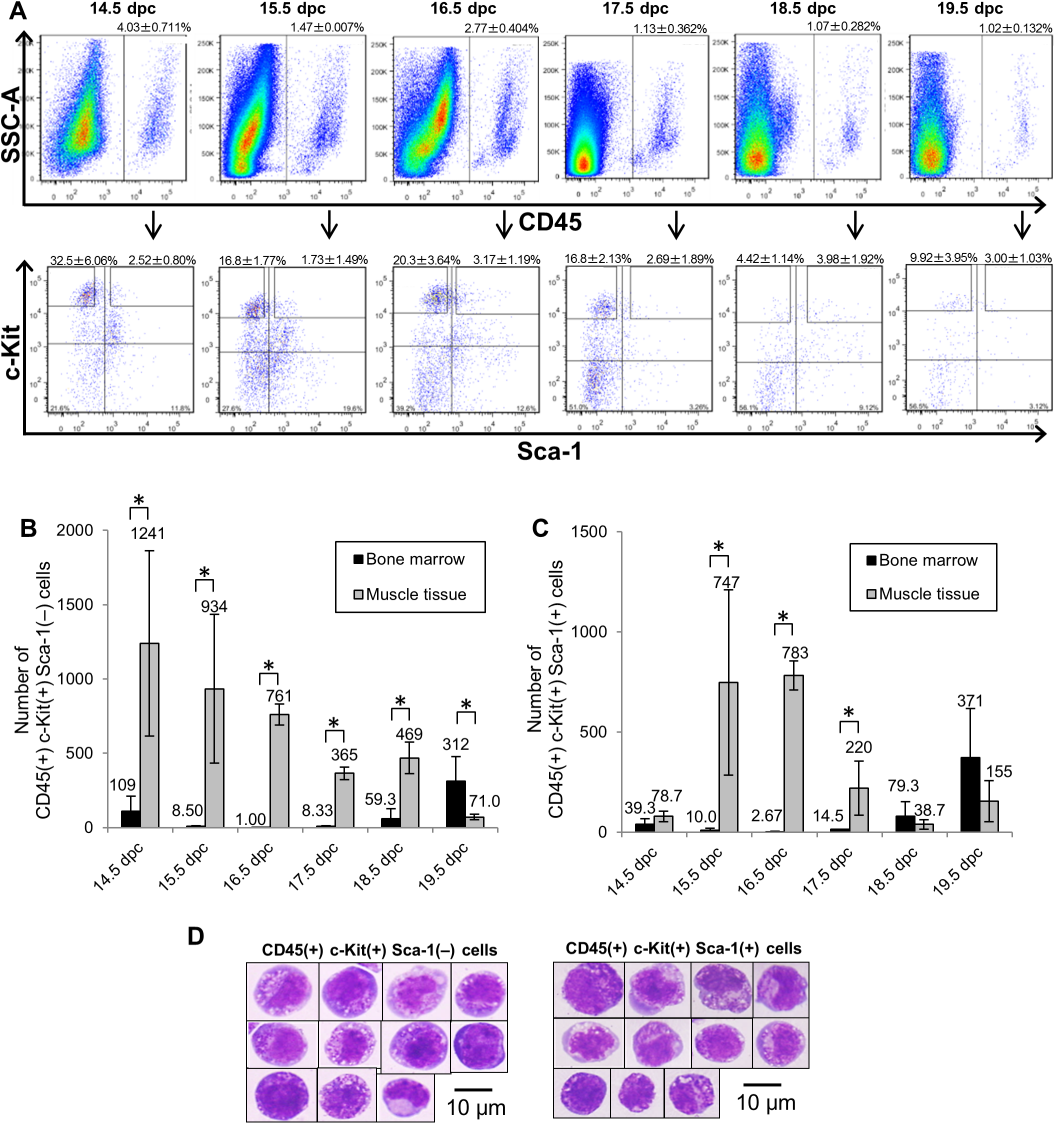
Flow cytometric analysis of hematopoietic cells in fetal muscle tissue. Single cell suspensions of fetal muscle tissue surrounding both left and right femurs at stages 14.5 to 19.5 dpc, as analyzed by flow cytometry. (A) Surface phenotypes of fetal muscle cells. Among CD45(+) cells (upper panels), surface expression of Sca-1 and c-Kit was analyzed (lower panels). (B) The number of CD45(+) c-Kit(+) Sca-1(–) cells per two femurs and per two sets of femur-surrounding muscle tissue at 14.5 to 19.5 dpc. Bars represent mean values and SD of three individual experiments. (C) The number of CD45(+) c-Kit(+) Sca-1(+) cells per two femurs and per two sets of femur-surrounding muscle tissue at 14.5 to 19.5 dpc. Bars represent mean values and SD of three individual experiments. (D) Morphology of CD45(+) c-Kit(+) Sca-1(–) cells (left) and CD45(+) c-Kit(+) Sca-1(+) cells (right) from 16.5 dpc muscle. Cells are stained with May-Grünwald Giemsa solution. Scale bar represents 10 μm for all panels. *p<0.01.

To further investigate surface marker expression of c-Kit(+) cells, we performed flow cytometric analysis of single cell suspensions of muscle tissues prepared from 14.5 to 19.5 dpc embryos using the pan-leukocyte marker CD45 (also expressed on fetal HPCs) [24], the HPC marker c-Kit, and the HSC marker Sca-1 [25]. CD45(+) c-Kit(+) cells were fractionated into 2 subpopulations based on Sca-1 expression, an analysis enabling us to distinguish HSCs from HPCs (Figs 2A and S3) and BM (S4 Fig) [26, 27]. To investigate the kinetics of CD45(+) c-Kit(+) Sca-1(+) cells compared to CD45(+) c-Kit(+) Sca-1(–) cells in embryonic muscle tissue and BM, we determined the total number of cells in these subpopulations per two femurs and per two sets of femur-surrounding muscle tissue at mid- to late-gestation (Fig 2B). From 14.5 to 19.5 dpc, the number of CD45(+) c-Kit(+) Sca-1(–) cells decreased 17.5-fold in muscle tissue (14.5 dpc: 1241±624 cells; 19.5 dpc: 71.0±17.5 cells), while it increased 2.9-fold in BM (14.5 dpc: 109±100 cells; 19.5 dpc: 312±166 cells) (Fig 2B). During this period, CD45(+) c-Kit(+) Sca-1(–) cells were more abundant in muscle tissue than in BM, while they became more abundant in BM by 19.5 dpc. The number of CD45(+) c-Kit(+) Sca-1(+) cells in muscle tissue peaked at 15.5–16.5 dpc and declined gradually by 19.5 dpc (Fig 2C). Likewise, the number of CD45(+) c-Kit(+) Sca-1(+) cells in BM gradually increased and peaked at 19.5 dpc, suggesting that BM hematopoiesis had been initiated at this stage. This trend was similar to one reported by Christensen et al, showing that long-term repopulating (LTR)-HSC activity is first detected in BM at 17.5 dpc [15]. To further characterize muscle HCs at 16.5 dpc, we analyzed surface marker expression by flow cytometry. We observed that muscle HCs express other hematopoietic surface markers, such as CD34 (4.22±2.75%), CD150 (2.85±1.34%) and EPCR (6.31±2.96%), at lower levels than do FL HPCs (S5A and S5B Fig). Among CD45(+) c-Kit(+) Sca-1(+) cells from muscle tissue, 62.8±13.8% were negative for lineage markers at 16.5 dpc, while in BM, 86.6±2.11% were negative for lineage markers at 19.5 dpc (S5C and S5D Fig).

To examine cell morphology, CD45(+) c-Kit(+) Sca-1(–) cells and CD45(+) c-Kit(+) Sca-1(+) cells were sorted from 16.5 dpc muscle tissues, stained with May-Grünwald Giemsa solution, and observed by microscopy. Both populations of cells appeared morphologically immature, as defined by a high nucleus/cytoplasm ratio and blue-colored cytoplasm (Fig 2D). Overall, these findings indicate that CD45(+) c-Kit(+) HCs localize in muscle surrounding bone and are more abundant in muscle than in BM between 14.5 and 17.5 dpc.

### Muscle hematopoietic cells express hematopoietic transcription factors

**Fig 3:**
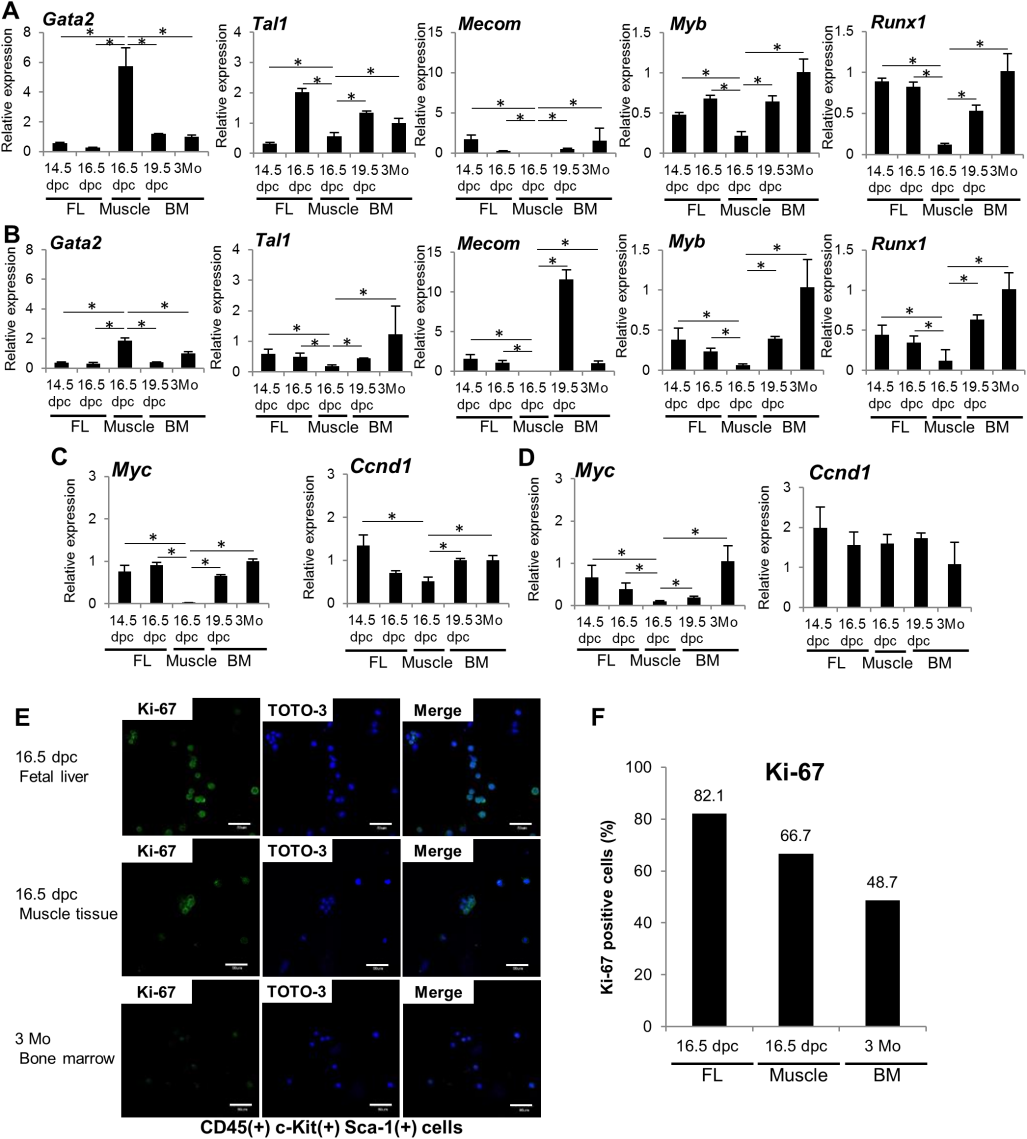
Expression of hematopoietic transcription factors and proliferation-related genes in muscle hematopoietic cells. RNA was extracted from sorted 14.5 dpc and 16.5 dpc FL CD45(+) c-Kit(+) Sca-1(–) and CD45(+) c-Kit(+) Sca-1(+) cells, 16.5 dpc muscle CD45(+) c-Kit(+) Sca-1(–) F4/80(–) and CD45(+) c-Kit(+) Sca-1(+) F4/80(–) cells, 19.5 dpc fetal BM CD45(+) c-Kit(+) Sca-1(–) and CD45(+) c-Kit(+) Sca-1(+) cells, and 3-month-old adult BM Lin(–) CD34(–) c-Kit(+) Sca-1(–) and CD45(+) c-Kit(+) Sca-1(+) cells, and expression of indicated factors assessed by real-time PCR. (A) Relative expression of hematopoietic transcription factors in the CD45(+) c-Kit(+) Sca-1(–) cell population in indicated samples. Each bar represents mean value and SD of three replicates. (B) Similar analysis in the CD45(+) c-Kit(+) Sca-1(+) cell population in indicated samples. Each bar represents mean value and SD of three technical replicates. (C) Relative expression of the proliferation-related genes *Myc* and *Ccnd1* in samples noted in (A). (D) Relative expression of proliferation-related genes in samples noted in (B). (E) Confocal images of Ki-67 in 16.5 dpc FL CD45(+) c-Kit(+) Sca-1(+) cells, 16.5 dpc muscle CD45(+) c-Kit(+) Sca-1(+) F4/80(–) cells, and 3-month-old adult BM Lin(–) CD34(–) c-Kit(+) Sca-1(+) cells. Shown is Ki-67 (green) and TOTO-3 iodide (blue) staining. Scale bar represents 50 μm for all panels. (F) The proportion of Ki-67(+) cells per total TOTO-3(+) cells in 16.5 dpc FL CD45(+) c-Kit(+) Sca-1(+) cells, 16.5 dpc muscle CD45(+) c-Kit(+) Sca-1(+) F4/80(–) cells, and 3-month-old adult BM Lin(–) CD34(–) c-Kit(+) Sca-1(+) cells. S5 Fig shows unstained Ki-67 control. *p<0.01.

Next, we examined expression of genes encoding hematopoietic transcription factors, such as *Gata2*, *Tal1*, *Mecom* (also known as *Evi-1*), *Myb* and *Runx1*, in CD45(+) c-Kit(+) Sca-1(–) and CD45(+) c-Kit(+) Sca-1(+) cells from FL at 14.5 and 16.5 dpc, from muscle tissue at 16.5 dpc, from BM at 19.5 dpc, and from Lin(–) CD34(–) c-Kit(+) Sca-1(–) and Lin(–) CD34(–) c-Kit(+) Sca-1 (+) cells from BM of 3-month-old mice (Fig 3A and 3B). All samples expressed *Gata2*, *Tal1*, *Myb* and *Runx1*. Muscle CD45(+) c-Kit(+) Sca-1(–) cells expressed higher levels of *Gata2* mRNA than did 14.5 dpc FL (10-fold higher, *p* = 0.0044), 16.5 dpc FL (21-fold higher, *p* = 0.0036), 19.5 dpc BM (4.7-fold higher, *p* = 0.0072) or 3-month-old adult BM (5.7-fold higher, *p* = 0.0062) cells (Fig 3A). Muscle CD45(+) c-Kit(+) Sca-1(+) cells also expressed higher levels of *Gata2* mRNA than did 14.5 dpc FL (4.9-fold higher, *p* = 0.00024), 16.5 dpc FL (5.8-fold higher, *p* = 0.00026), 19.5 dpc BM (4.8-fold higher, *p* = 0.00026), or 3-month-old adult BM (1.9-fold higher, *p* = 0.039) cells (Fig 3B). By contrast, expression levels of *Tal1*, *Mecom*, *Myb*, and *Runx1* were lower in muscle CD45(+) c-Kit(+) Sca-1(–) and CD45(+) c-Kit(+) Sca-1(+) cells than in comparable cells in other fractions. In addition, neither CD45(+) c-Kit(+) Sca-1(–) nor CD45(+) c-Kit(+) Sca-1(+) muscle cells expressed *Mecom* mRNA. When we also assessed relative expression of hematopoietic transcription factors using comparative Ct methods, we found that muscle CD45(+) c-Kit(+) Sca-1(–) cells and CD45(+) c-Kit(+) Sca-1(+) cells expressed the highest *Gata2* mRNA levels of any hematopoietic transcription factor gene analyzed (S6 Fig). Taken together, relative to FL or BM cells, HCs at 16.5 dpc muscle exhibited higher *Gata2* expression but lower expression of *Tal1*, *Mecom*, and *Myb*.

### Hematopoietic cells found in muscle tissue proliferate more slowly than do FL cells

We next assessed proliferative status of muscle CD45(+) c-Kit(+) Sca-1(–) and CD45(+) c-Kit(+) Sca-1(+) cells at 16.5 dpc based on real-time PCR analysis of *Myc* and *Ccnd1* expression (Fig 3C and 3D). *Myc* transcript levels in muscle CD45(+) c-Kit(+) Sca-1(–) cells at 16.5 dpc were significantly lower than those seen in 14.5 dpc FL (25.5-fold lower, *p* = 0.026), 16.5 dpc FL (30.5-fold lower, *p* = 0.023), 19.5 dpc BM (22.0-fold lower, *p* = 0.0069) or 3-month-old adult BM cells (33.5-fold lower, *p* = 0.011). CD45(+) c-Kit(+) Sca-1(+) cells showed similarly decreased *Myc* expression relative to 14.5 dpc FL (6.90-fold lower, *p* = 0.00085), 16.5 dpc FL (4.00-fold lower, *p* = 2.2×10^−5^), 19.5 dpc BM (1.96-fold lower, *p* = 4.3×10^−6^), and 3-month-old adult BM (10.8-fold lower, *p* = 8.3×10^−6^). At 16.5 dpc, *Ccnd1* mRNA expression was lowest in muscle CD45(+) c-Kit(+) Sca-1(–) cells than in any other fraction (Fig 3C, right). However, we observed no significant differences in *Ccnd1* mRNA expression in CD45(+) c-Kit(+) Sca-1(+) cells compared to any other tissues analyzed (Fig 3D, right). We also performed immunocytochemistry using antibodies against Ki-67, which marks proliferative (G1, S, G2 and M phase) cells but is absent in G0 phase [28, 29] (Figs 3E and S7). The proportion of Ki-67(+) cells was 82.1% of CD45(+) c-Kit(+) Sca-1(+) cells in FL at 16.5 dpc, 66.7% of CD45(+) c-Kit(+) Sca-1(+) cells in muscle at 16.5 dpc and 48.7% of Lin(–) CD34(–) c-Kit(+) Sca-1(+) cells in 3-month-old adult BM, suggesting that CD45(+) c-Kit(+) Sca-1(+) cells in muscle at 16.5 dpc are less proliferative than are HCs in FL (Fig 3F).

### Muscle CD45(+) c-Kit(+) cells possess colony forming capacity

**Fig 4:**
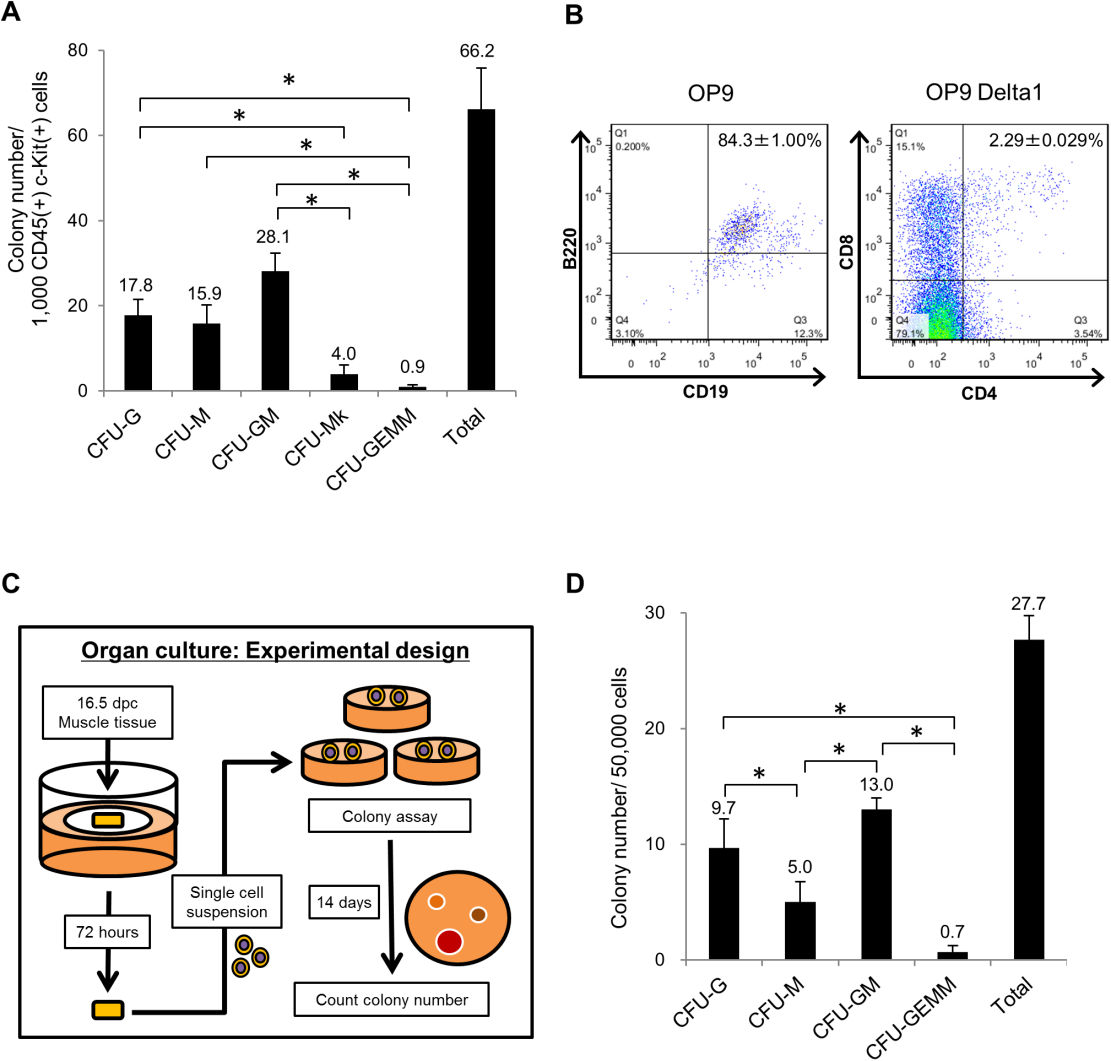
Colony forming capacity of muscle CD45(+) c-Kit(+) cells. Muscle CD45(+) c-Kit(+) cells exhibit hematopoietic activity. (A) One thousand sorted muscle CD45(+) c-Kit(+) cells at 16.5 dpc were cultured in semisolid medium containing stem cell factor (SCF), interleukin (IL)-3, IL-6 and erythropoietin (Epo). On day 14, the number of CFU-G (colony-forming units of granulocytes), CFU-M (of macrophages), CFU-GM (of granulocytes and macrophages), CFU-Mk (of megakaryocytes), and CFU-GEMM (of granulocytes, erythrocytes, monocytes and macrophages) and the total colony number were counted. Bars represent means and SD of three culture dishes. (B) Fetal muscle CD45(+) c-Kit(+) cells obtained at 16.5 dpc were co-cultured with OP9 or OP9 Delta1 lines to assess lymphoid potential. Shown are surface expression of CD19 and B220 (B lymphoid markers) on cells cultured with OP9 cells for 16 days (left panel) and surface expression of CD4 and CD8 (T lymphoid markers) on cells cultured with OP9 Delta1 cell line for 16 days (right panel). (C) Experimental design of the colony formation assay after an organ culture step. Muscle tissue at 16.5 dpc was cultured on filter paper for 72 hours and a single cell suspension was prepared. One thousand cells were cultured and evaluated as in (A). (D) Shown are the number of cells of each colony type and total number of colonies on day 14. *p<0.01.

To evaluate their hematopoietic potential, we sorted CD45(+) c-Kit(+) muscle cells at 16.5 dpc and performed colony formation assays. To do so, we seeded 1,000 sorted cells in a 35-mm dish and determined the number of colonies on day 14. As shown in Fig 4A, muscle tissue CD45(+) c-Kit(+) cells formed hematopoietic colonies (Colony-Forming Units (CFU) of granulocytes (CFU-G), macrophages (CFU-M), granulocytes and macrophages (CFU-GM), megakaryocytes (CFU-Mk) and granulocyte, erythrocytes, monocytes and macrophages (CFU-GEMM)), suggesting that muscle CD45(+) c-Kit(+) cells contain HPCs. Among all, the number of CFU-GM was greatest. At day 21 of culture, we also counted high proliferative potential colony forming cells (HPP-CFCs), which can form large colonies *in vitro* and possess higher hematopoietic potential and observed that muscle CD45(+) c-Kit(+) cells generated 2 HPP-CFCs per 1,000 cells. In addition, we performed co-culture of muscle CD45(+) c-Kit(+) cells with OP9/OP9 Delta1 cell lines to assess their lymphoid potential. As shown in Fig 4B, on culture day 16, muscle CD45(+) c-Kit(+) cells cultured with the OP9 line expressed B lymphoid markers CD19 and B220 (84.3%±1.00%), while those cultured with the OP9 Delta1 line expressed the T lymphoid markers CD4 and CD8 (2.29±0.029%). These results suggest that muscle CD45(+) c-Kit(+) cells can differentiate into both myeloid and lymphoid lineages.

Next, we evaluated the influence of the circulation on embryonic muscle tissue using organ culture [5]. Using the experimental design shown in Fig 4C, we performed colony formation assays after 72 hours of organ culture, and 50,000 cells were seeded per dish. After 72 hours we assessed the surface phenotype of cells and both CD45(+) c-Kit(+) Sca-1(+) and CD45(+) c-Kit(+) Sca-1(–) cells were observed (S8 Fig). At the end of the culture period, we found that 60.5% of cells incorporated propidium iodide (a marker of dead cells), and 172 cells among 50,000 cells of muscle after culture expressed both CD45 and c-Kit. On day 14, we observed formation of hematopoietic colonies (27.7±2.08 colonies) consisting of CFU-G (9.70±2.52 colonies), CFU-M (5.00±1.73 colonies), CFU-GM (13.0±1.00) and CFU-GEMM (0.70±0.58 colonies) (Fig 4D). Overall, colony formation analysis revealed that muscle HCs possesses HPC activity.

### Fetal CD45(+) c-Kit(+) cells migrate from liver to muscle to BM

**Fig 5:**
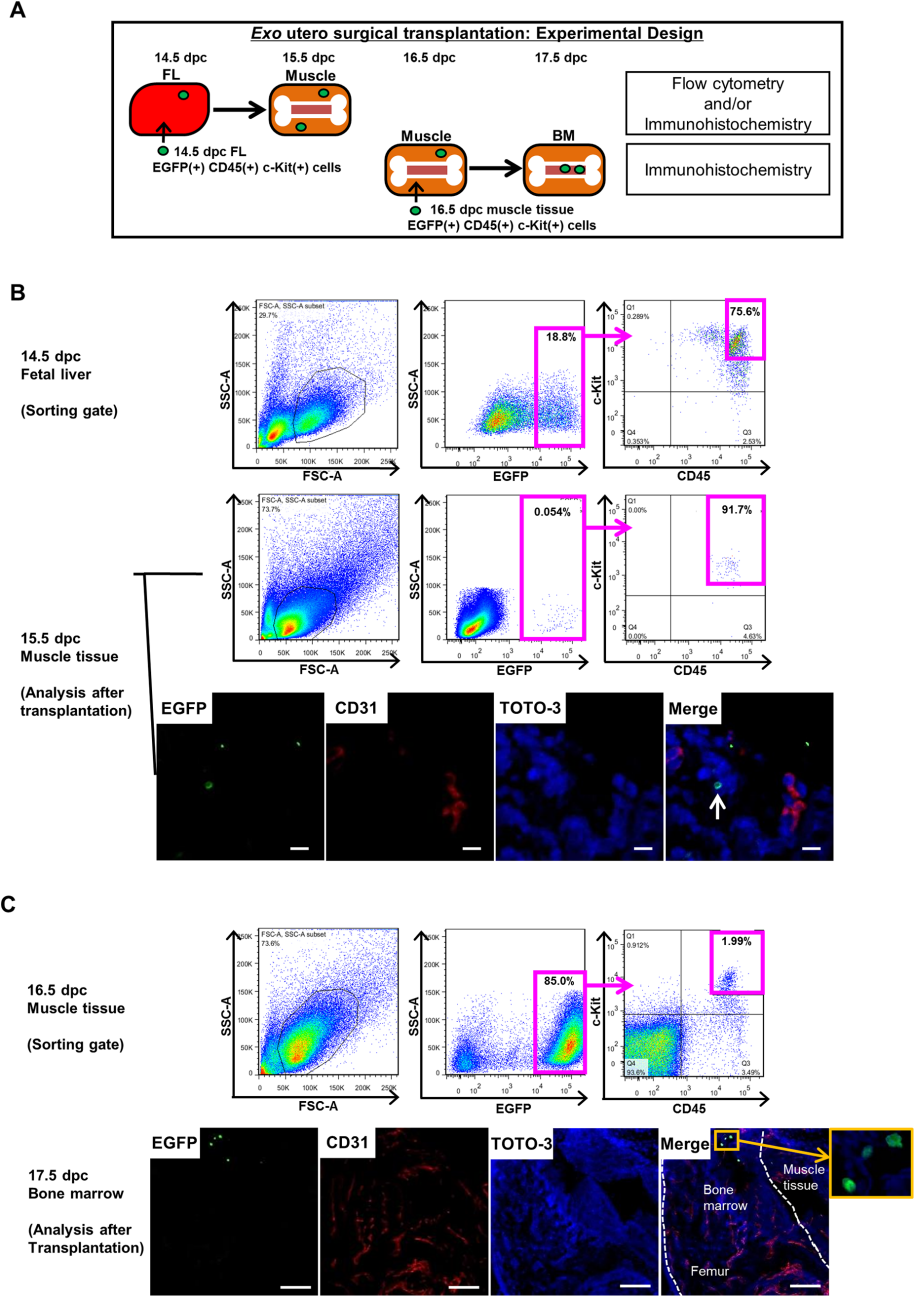
Fetal CD45(+) c-Kit(+) cells migrate from liver to muscle and then to BM. Fetal CD45(+) c-Kit(+) cell migration was assessed by *exo utero* surgical transplantation. (A) Experimental design showing the transplantation protocol. EGFP(+) CD45(+) c-Kit(+) cells were sorted from FL of EGFP Tg mouse embryos at 14.5 dpc and from muscle tissues surrounding femurs at 16.5 dpc and transplanted into corresponding tissues of recipient C57BL/6 mouse embryos at the same developmental stage. After 24 hours, the presence of EGFP(+) cells in muscle tissue and BM was analyzed by flow cytometry and/or immunohistochemistry. (B) Flow cytometric profile exhibiting gate setting used to sort EGFP(+) CD45(+) c-Kit(+) cells from FL of EGFP Tg embryos at 14.5 dpc (upper). At 24 hours after transplantation, muscle tissue was dissociated into single cells and analyzed by flow cytometry. Representative flow cytometric profile shows EGFP(+) CD45(+) c-Kit(+) cells present in muscle tissue of 15.5 dpc recipients (middle). Muscle tissue was sectioned and immunostained with CD31 (red) and TOTO-3 iodide (blue). Representative confocal image showing EGFP(+) donor cells (arrow; green) in muscle (lower). Scale bar represents 10 μm for all panels. (C) Flow cytometric profile exhibiting gate setting used to sort EGFP(+) CD45(+) c-Kit(+) cells from muscle tissue of EGFP Tg embryos at 16.5 dpc (upper). At 24 hours after transplantation, femurs were sectioned and immunostained with CD31 (red) and TOTO-3 iodide (blue). Scale bar represents 100 μm. EGFP(+) donor cells (green) are present in BM at 17.5 dpc. Boxed area of merge panel is shown at higher magnification.

To investigate migration of fetal CD45(+) c-Kit(+) cells, we sorted EGFP(+) CD45(+) c-Kit(+) cells from FL of EGFP transgenic (Tg) mouse embryos at 14.5 dpc or from muscle tissue of Tg embryos at 16.5 dpc and transplanted them into corresponding tissues of C57BL/6 recipient embryos using *exo utero* surgical techniques [30]. After 24 hours, we undertook flow cytometry and/or immunohistochemistry to monitor EGFP(+) donor cells in muscle tissue or BM, based on the procedure shown in Fig 5A. EGFP(+) CD45(+) c-Kit(+) cells were sorted from FL for transplantation (Fig 5B, upper). Flow cytometric analysis showed that EGFP(+) CD45(+) c-Kit(+) cells were present in muscle tissue of 15.5 dpc recipients (Fig 5B, middle). Among EGFP(+) CD45(+) c-Kit(+) cells, 17.6±1.13% were Sca-1(+) and 82.4±1.13% were Sca-1(–) (S9A Fig). We also assessed expression of hematopoietic transcription factors in EGFP(+) donor cells 24 hours after transplantation. EGFP(+) donor cells that had migrated expressed *Gata2*, *Tal1*, *Myb* and *Runx1* but not *Mecom* (S9B Fig). Transplanted cells were also observed in other areas of muscle tissue that surround the tibia and humerus (S9C Fig). Immunostaining of muscle tissue surrounding femurs also revealed the presence of EGFP(+) donor cells (Fig 5B, lower). We then sorted EGFP(+) CD45(+) c-Kit(+) cells from muscle for transplantation into recipient muscle (Fig 5C, upper). Immunostaining of femur showed that EGFP(+) donor cells were present in BM (Fig 5C, lower). EGFP(+) donor cells also remained in muscle tissue in all recipients (data not shown). We further investigated migration of muscle CD45(+) c-Kit(+) cells from FL to fetal BM by tracking cells for up to 72 hours. As shown in S9D and S9E Fig, Qdot585-labeled donor cells transplanted into FL were detected in BM 72 hours later, indicating that they had migrated into fetal BM. Table 1 summarizes data relevant to *exo utero* surgery transplantation. Taken together, these observations indicate that during embryogenesis CD45(+) c-Kit(+) cells migrate from FL to muscle tissue and thereafter move from muscle tissue to BM.

**Table 1:** Summary of *exo utero* surgical transplantation data.

Donor cell source	No. of recipients (No. of injected cells/recipient)	Survival rate after 24 hours	No. of recipients exhibiting donor cells (Number of donor cells detected)
**14.5 dpc FL**	13 (1.4x10^5^ cells/recipient)	76.9%	10 (8–123 cells/muscle tissue) (1–20 cells/2 femurs)
**16.5 dpc muscle tissue**	8 (5.0x10^3^ cells/recipient)	100%	4 (1–4 cells/2 femurs)

We then sorted EGFP(+) CD45(+) c-Kit(+) cells of EGFP transgenic mouse embryos from FL at 14.5 dpc and from muscle tissue at 16.5 dpc and transplanted cells into corresponding tissues of C57BL/6 mouse recipients utilizing *exo utero* transplantation. In some experiments, CD45(+) c-Kit(+) cells were sorted from FL of C57BL/6 mouse embryos at 14.5 dpc and labeled with Qdot585 dye. After 24 hours, the presence of EGFP(+) or Qdot585(+) cells was evaluated using flow cytometry and/or immunohistochemistry. Transplantation results are summarized.

## Discussion

Here, we demonstrate that HPCs reside in embryonic muscle tissue before BM hematopoiesis. These muscle HPCs migrate from FL and move to the BM, suggesting that HPC homing occurs earlier than previously reported and that these HPCs reside in muscle for a period as they make their migration.

Two groups have reported that adult mouse skeletal muscle contains cells with HSPC potential [31, 32]. Based on flow cytometry, they found CD45(–) Sca-1(+) c-Kit(–) and CD45(–) Sca-1(+) c-Kit(+) cells located in what is known as the side population, in which cells show high Hoechst 33342 dye efflux activity. In our study, we sorted CD45(+) c-Kit(+) Sca-1(–) and CD45(+) c-Kit(+) Sca-1(+) cells from embryonic muscle, suggesting that they are HCs based on CD45 expression. These cells are distinct from the CD45(–) Sca-1(+) c-Kit(–) and CD45(–) Sca-1(+) c-Kit(+) cells of adult muscle, which are likely multi-potent stem cells with the capacity to differentiate or trans-differentiate into hematopoietic lineages. Others have demonstrated that CD45(+) Sca-1(+) cells derived from adult muscle possess HPC activity but do not express c-Kit [33]. When those authors performed a colony formation assay using 200,000 adult muscle cells, Sca-1(–) and Sca-1(+) cells generated approximately 60 and 30 hematopoietic colonies, respectively. In our study, we focused on CD45(+) c-Kit(+) HCs in mouse embryos. When we performed colony formation assays using 1,000 embryonic muscle cells at 16.5 dpc, CD45(+) c-Kit(+) Sca-1(–) and CD45(+) c-Kit(+) Sca-1(+) cells generated 60.0±9.5 and 37.3±2.5 hematopoietic colonies, respectively, suggesting an enrichment of HPCs due to either CD45 and c-Kit expression and/or higher hematopoietic activity. As shown in S2B Fig, adult muscle tissue exhibits only a few CD45(+) Sca-1(+) cells and no CD45(+) c-Kit(+) cells, findings in agreement with an earlier study [33]. When we transplanted CD45(+) Sca-1(+) cells into recipient muscle tissue, some cells remained at the transplant site for 24 hours, implying that some embryonic muscle HPCs are ancestors of adult muscle HPCs, despite the fact that adult CD45(+) Sca-1(+) cells lack c-Kit expression (Fig 5).

The fetal blood circulation system becomes functional by 14.5 dpc and is a potential source of HCs [15]. To exclude the possibility that blood contamination is the source of embryonic muscle HPCs observed here, we performed CD45, c-Kit and CD31 immunostaining of serial sections of muscle tissues at 14.5 dpc and 16.5 dpc (Fig 1C) and found that most CD45(+) c-Kit(+) cells were localized in muscle tissue and their absolute number was greater outside of blood vessels in muscle tissue than in inside vessels. (Fig 1D). We also performed colony formation assays with muscle tissue cells after 72 hours of organ culture to exclude the influence of circulation. That analysis confirmed the existence of HPCs in muscle tissues at 16.5 dpc (Figs 4C and 4D and S8). Christensen et al previously reported that in mice HSCs are present in fetal blood at low but constant levels during late gestation [15]. Likewise, our flow cytometry analysis indicates that fetal blood contains CD45(+) c-Kit(+) cells but at a low frequency (7.41x10^-4^±5.94x10^-4^% of living cells) at 16.5 dpc (Figs 1C and 1D and S10). We conclude that muscle HPCs observed here are not likely due to contamination by blood.

Gene expression analysis indicated that HPCs in muscle express hematopoietic transcription factor genes such as *Gata2*, *Tal1*, *Myb* and *Runx1*, but (with the exception of *Gata2)* at levels lower than in HPCs in other tissues. This finding implies that CD45(+) c-Kit(+) cells in embryonic muscle possess decreased hematopoietic potential, although they show similar surface marker expression and are potentially in a transition stage between cells leaving the FL and those in the BM. To assess their hematopoietic potential, we performed colony formation assays. Previously, we reported that CD31(+) CD34(+) c-Kit(+) cells derived from intra-aortic clusters at 10.5 dpc generated 113.4 colonies per 1,000 cells; here, we observed that 1,000 CD45(+) c-Kit(+) cells from 16.5 dpc muscle gave rise to 66.2 colonies, indicative of a relative decrease in hematopoietic potential [34]. Interestingly, muscle HPCs lack expression of *Mecom*, an oncogenic transcription factor regulating embryonic HPC activity [35]. *Mecom* knockout mice exhibit impaired hematopoietic and vascular development and die at 10.5 dpc [36]. The availability of *Mecom*-IRES-GFP reporter mice allows fractionation by flow cytometry of BM cells negative for lineage markers and positive for Sca-1 and c-Kit (LSK cells) as GFP(+) (showing high *Mecom* expression) and GFP(–) (showing low or no *Mecom* expression) cells [35]. Colony formation assays showed that sorted GFP(+) and GFP(–) LSK cells generated hematopoietic colonies, but that a greater number was generated from GFP(+) cells [35]. This observation suggests that *Mecom* expression levels define hematopoietic potential and supports the idea that CD45(+) c-Kit(+) cells in muscle exhibit decreased hematopoietic potential. Low *Myc* expression in muscle HPCs reported here also implies decreased hematopoietic potential and a more quiescent status, as Myc stimulates cell proliferation and controls the balance of self-renewal activity, quiescence and differentiation [37–39]. An interesting exception is our observation that muscle HPCs express *Gata2* at higher levels than do HPCs from other tissues. When we quantified *Gata2* expression using the comparative Ct method, *Gata2* was the most highly expressed hematopoietic transcription factor in muscle HPCs (S6 Fig). *Gata2* knockout embryos die by 11.5 dpc due to severe anemia, and cells from their YS and AGM regions exhibit relatively low hematopoietic potential, as assessed by colony formation [40]. *Gata2* deletion in VE-cadherin expressing cells decreases the number of HPCs in the YS, AGM region and FL and impairs intra-aortic cluster formation. On the other hand, *Gata2* overexpression in mouse BM HSCs decreases the number of both CFU-C and CFU-S and impairs HSC activity [41]. *Myb* knockout embryos also die around 15.5 dpc due to severe anemia [42], and *Myb* overexpression in mouse ES cells impairs hematopoietic differentiation *in vitro* [43]. Thus, decreased hematopoietic potential observed in muscle HPCs might be due to the relative expression of *Gata2* (high) to *Myb* (low).

It remains unclear why migrating HPCs transiently reside in muscle. Nonetheless, exposure to an embryonic muscle environment may be required to modulate cells’ hematopoietic potential, possibly through signals that regulate appropriate levels of hematopoietic transcription factors. Between 10 and 15 dpc, FL actively develops and is colonized by HCs to become a major hematopoietic organ [44]. Then, as embryos develop and hepatogenesis becomes more active between 15 dpc and post-natal stages, the FL hematopoietic compartment likely becomes smaller. As the FL environment becomes rich in cytokines and ECM proteins that accelerate hematopoietic differentiation, HPCs may exit the FL to avoid stimulation by hematopoietic differentiation factors. Since the vascular structure of fetal BM is not yet well-developed at mid-gestation [45], BM may not constitute an environment that can sustain hematopoietic cells at 16.5 dpc, implying that HPCs require other environments to pause. Given that HPCs survive during an organ culture step (Fig 4D), an alternate hypothesis is that muscle tissues secrete factors required to sustain HPCs at 16.5 dpc.

Homing mechanisms used by HCs to move from FL to fetal BM are not fully understood. Here, we used an *exo utero* technique to show that HPCs found in muscle tissue surrounding bones migrate from FL to muscle and then to BM (Fig 5). Future analysis should address developmental implications of this migration to expand our knowledge of how HCs are regulated.

## Supporting Information

S1 Checklist (PDF)

S1 Fig Analysis of c-Kit(+) cells in bone marrow and muscle tissue surrounding humerus. (TIF)

S2 Fig Analysis of c-Kit(+) cells in BM and muscle tissue surrounding femurs in adult mice. (TIF)

S3 Fig Unstained control for muscle tissue flow cytometric analysis. (TIF)

S4 Fig Flow cytometric analysis of hematopoietic cells in fetal BM. (TIF)

S5 Fig Surface marker expression of muscle hematopoietic cells. (TIF)

S6 Fig Hematopoietic transcription factor gene expression in muscle hematopoietic cells. (TIF)

S7 Fig Unstained Ki-67 immunocytochemistry control. (TIF)

S8 Fig Flow cytometric analysis of muscle tissue after 72 hours of organ culture. (TIF)

S9 Fig *Exo utero* cell transplantation analysis. (TIF)

S10 Fig Flow cytometric analysis of fetal blood. (TIF)
