# Supplementary figures and images for: Embryonic Hematopoietic Progenitor Cells Reside in Muscle before Bone Marrow Hematopoiesis
Source: PLoS One. 2015 Sep 21;10(9):e0138621. doi: 10.1371/journal.pone.0138621 (PMC4577119; doi:10.1371/journal.pone.0138621)

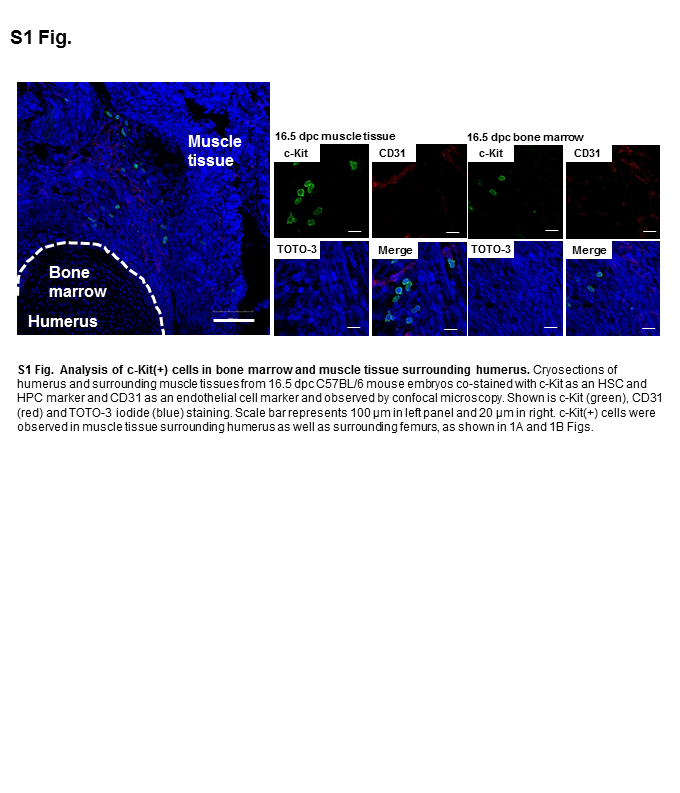

Supplement: S1 Fig — (TIF) [file pone.0138621.s002.TIF]

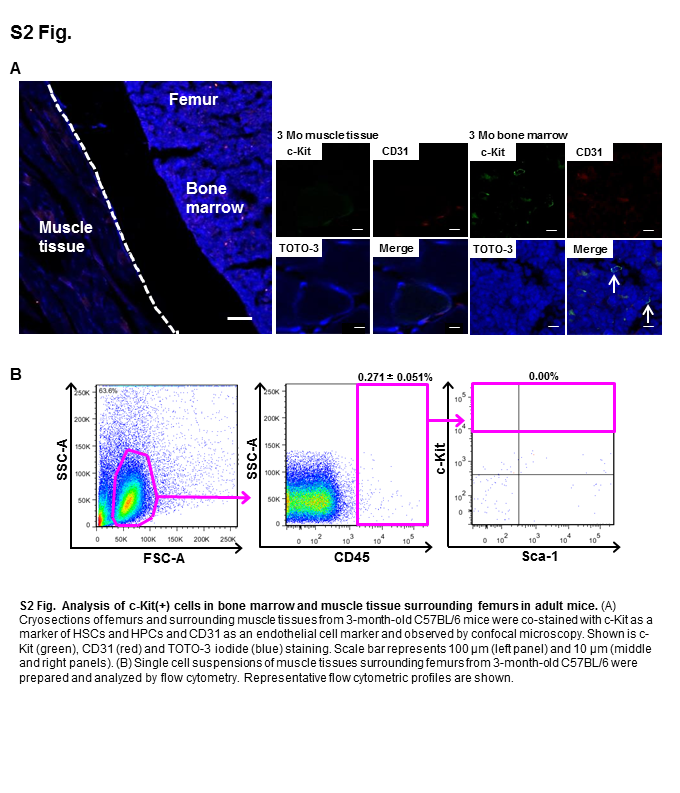

Supplement: S2 Fig — (TIF) [file pone.0138621.s003.TIF]

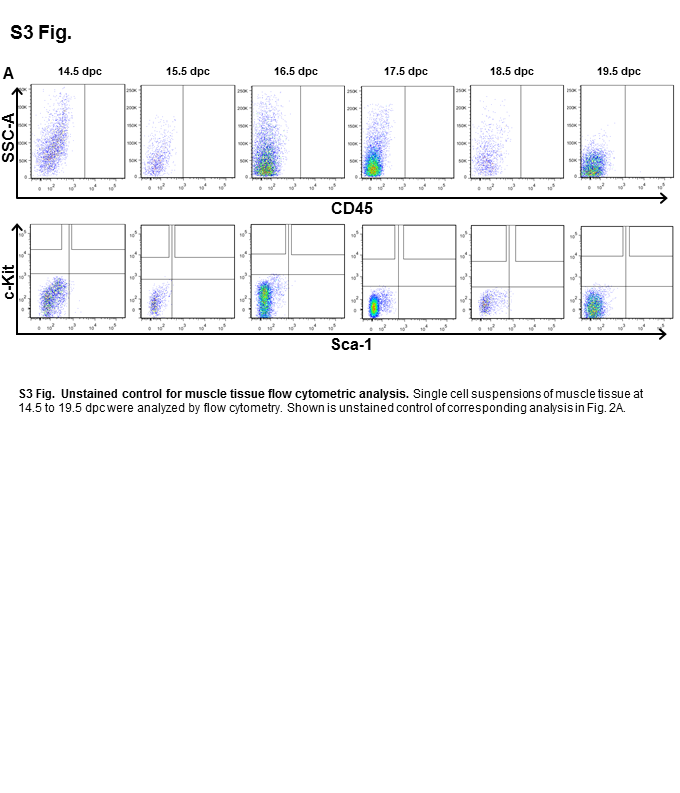

Supplement: S3 Fig — (TIF) [file pone.0138621.s004.TIF]

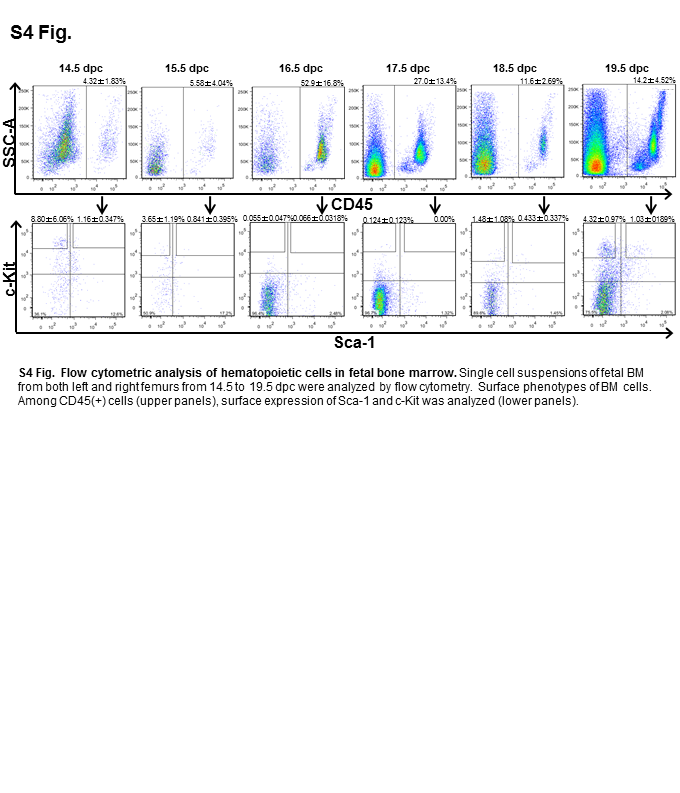

Supplement: S4 Fig — (TIF) [file pone.0138621.s005.TIF]

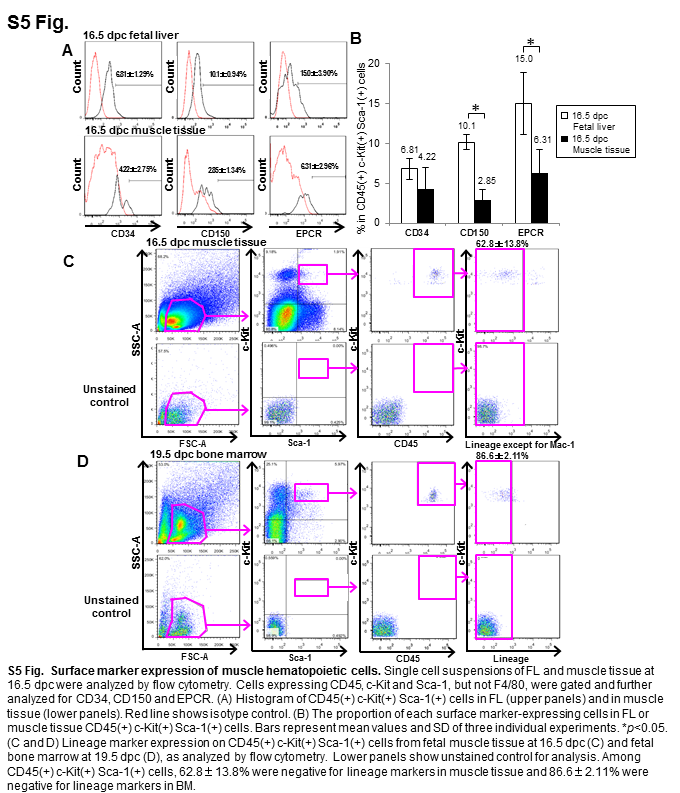

Supplement: S5 Fig — (TIF) [file pone.0138621.s006.TIF]

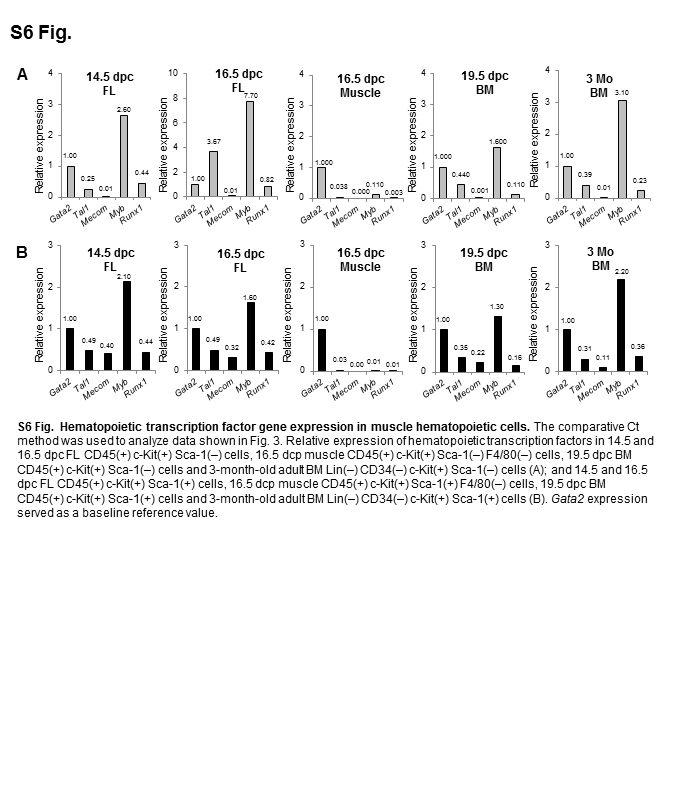

Supplement: S6 Fig — (TIF) [file pone.0138621.s007.TIF]

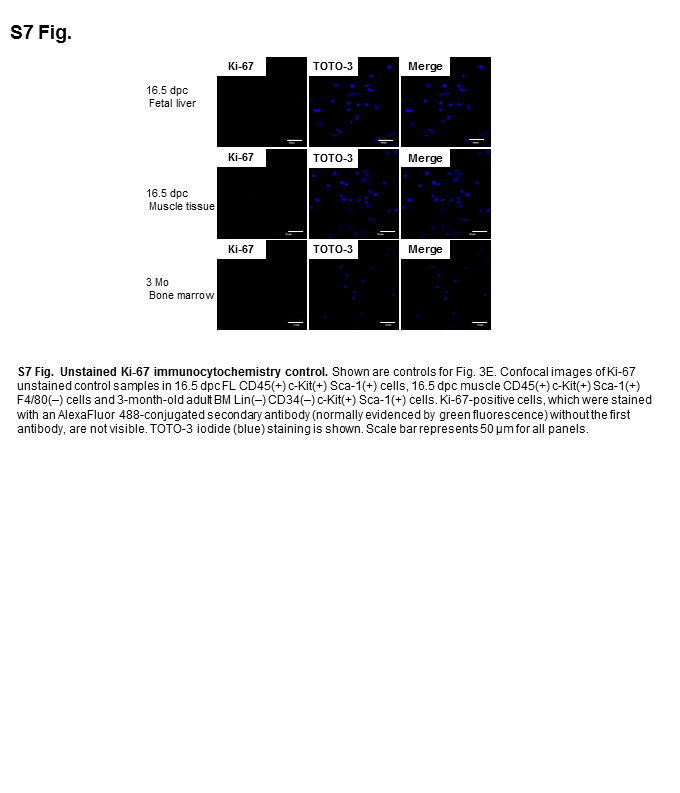

Supplement: S7 Fig — (TIF) [file pone.0138621.s008.TIF]

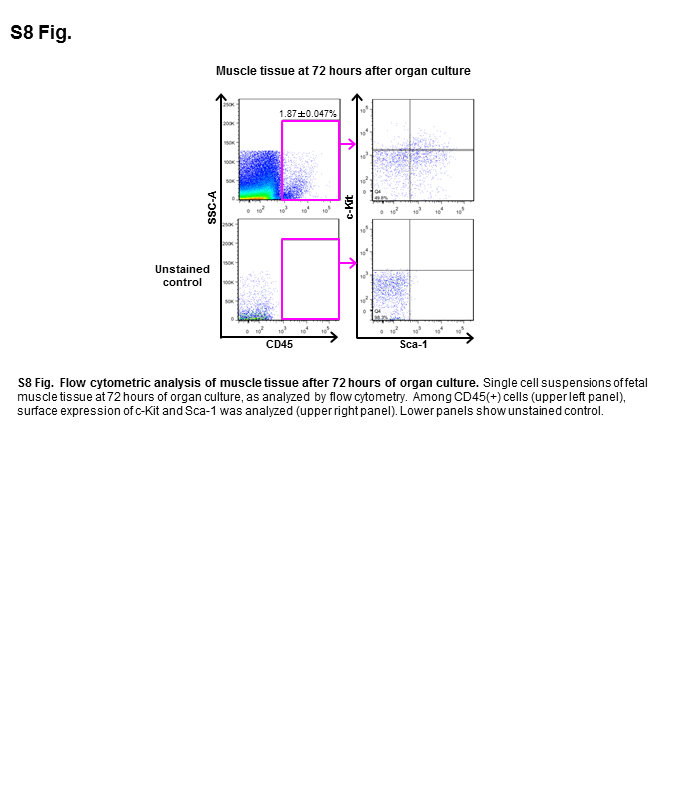

Supplement: S8 Fig — (TIF) [file pone.0138621.s009.TIF]

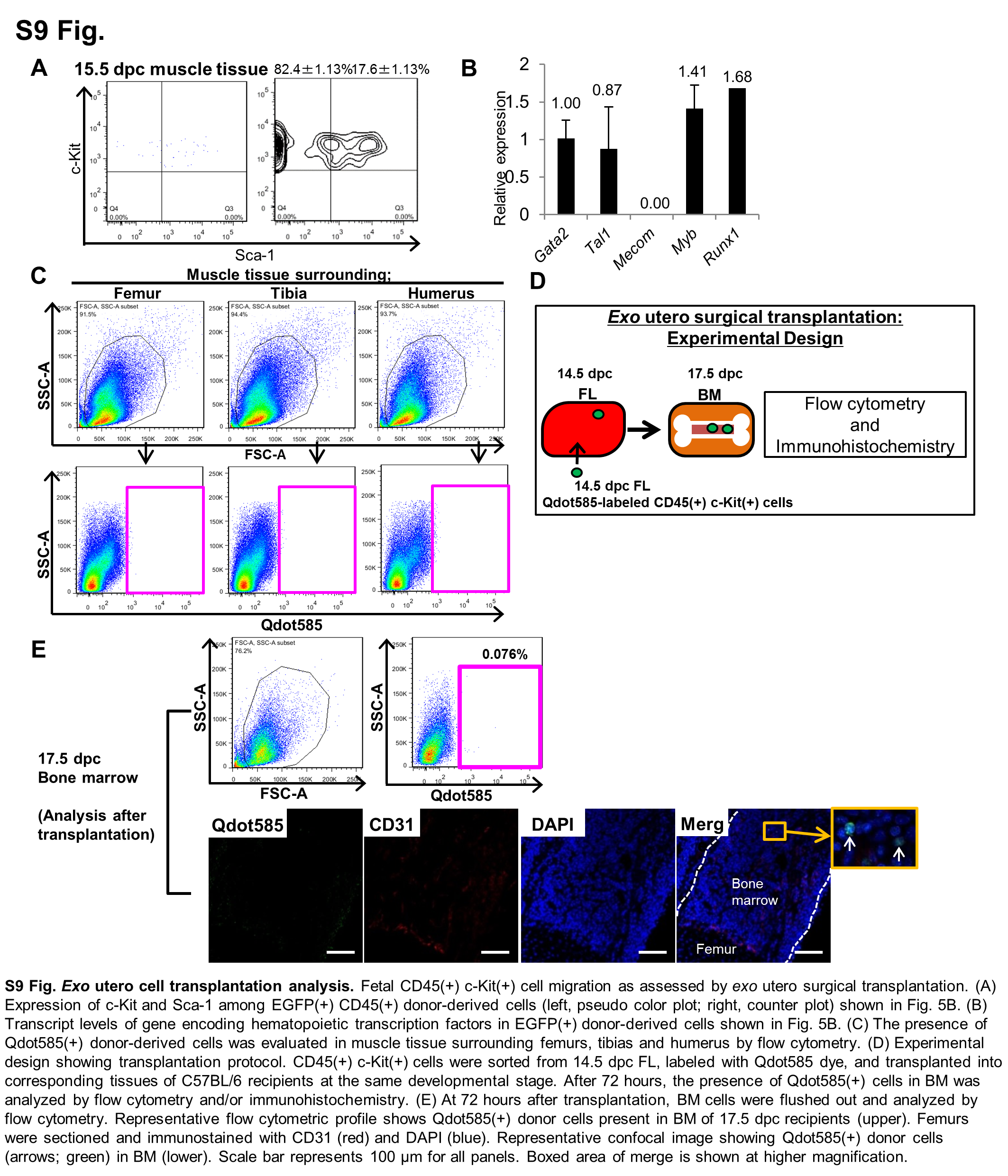

Supplement: S9 Fig — (TIF) [file pone.0138621.s010.TIF]

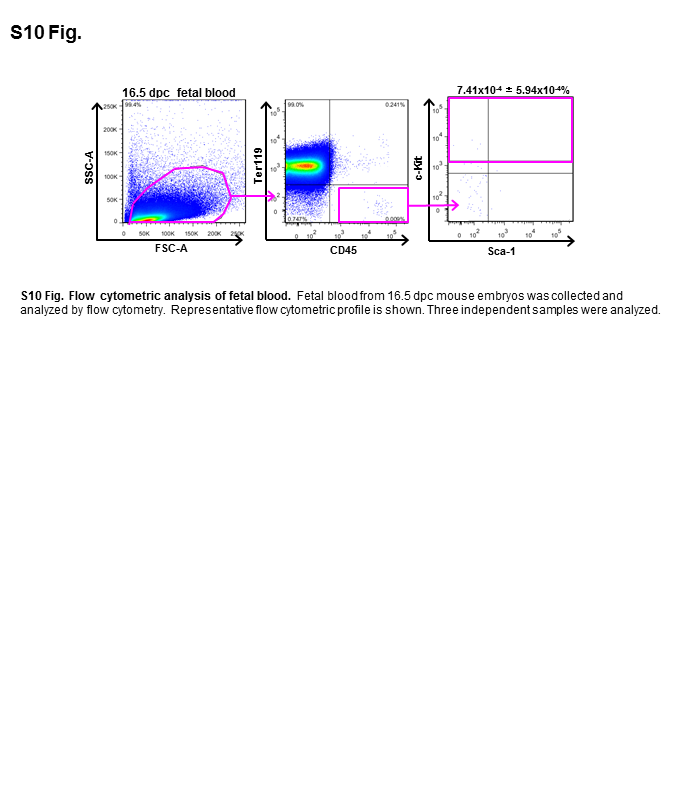

Supplement: S10 Fig — (TIF) [file pone.0138621.s011.TIF]
